# Association Between Ultra-Processed Food Consumption and Hypertension Incidence: Findings From RaNCD Cohort Project

**DOI:** 10.1155/ijhy/2495258

**Published:** 2025-04-15

**Authors:** Parsa Amirian, Mahsa Zarpoosh, Yahya Pasdar

**Affiliations:** ^1^Kermanshah University of Medical Science (KUMS), Kermanshah, Iran; ^2^Nutritional Sciences Department, School of Public Health, Kermanshah University of Medical Sciences, Kermanshah, Iran

**Keywords:** cohort, hypertension, survival analysis, ultra-processed foods

## Abstract

**Background:** Ultra-processed food (UPF) consumption is increasing rapidly due to large-scale food production. Being a public health issue, hypertension is affecting 1.28 billion adults globally. This study investigates the link between UPF consumption and hypertension.

**Methods:** We included 8150 participants at the risk of hypertension in the final analysis. UPF consumption was assessed using data from the available Food Frequency Questionnaire (FFQ), and the amount of UPF consumption of each participant in a day was assessed. Cox proportional models with covariates including age, sex, residence type, marital status, socioeconomic status, physical activity, familial history of hypertension, and fasting blood sugar were used to assess the association between UPF consumption and hypertension in the main model and sensitivity analysis. Age, residence type, and the third tertile of UPF interacted with time in our model, which was addressed accordingly.

**Results:** The mean participant age was 46.25 years (47.58% male) with a mean follow-up of 7.65 years. The mean daily UPF intake was 88.07 g. During follow-up, 862 hypertension cases were recorded. After adjusting the main model for confounders, the hazard ratios for the second and third tertiles of UPF consumption were 1.13 (95% CI, *p* value) (0.96–1.32, 0.13) and 0.65 (95% CI, *p* value) (0.46–0.91, 0.01), respectively, compared to the first tertile.

**Conclusion:** We found significant association between the third tertile of UPF intake and hypertension; moreover, we identified significant associations between hypertension incidence and some demographic factors, warranting further investigation.

## 1. Introduction

Since the introduction of NOVA food classification that separates foods and beverages into four categories, including unprocessed, processed culinary ingredients, processed, and ultra-processed foods (UPFs), the light was shed on an overlooked aspect of nutrition which is the journey that food takes from agricultural steps to when it is eaten [1]. Rural-to-urban migration, agrarian transformation, population growth, heavy UPF marketing, and economic progress, alongside many other factors, led to excessive UPF consumption percentages in the population diet; for example, population growth and the demand for food worldwide led the economy to produce food in large scales which are mostly UPFs; therefore, understanding the effects of UPF consumption in many aspects of health is crucial [2]. Being a major risk factor and health concern, as said by the World Health Organization (WHO), 1.28 billion adults suffer from hypertension (HTN); it is a substantial reason for premature death around the globe [3]. The HTN prevalence is not equal in different parts of the world; low and middle-income countries have a higher prevalence than developed countries, which makes HTN-related research in regions like the Middle East and North Africa much more critical [3]. To the best of our knowledge, the association between UPF consumption and HTN incidence, despite being a significant concern and area of interest, has been rarely investigated before, especially in low and middle-income countries. The UPF/HTN association was investigated before in countries like Spain and Brazil in a prospective cohort style with mixed results. Mendonça et al. used the Spanish Navarra Project data and found that participants in the highest tertile of UPF consumption had 21% higher chance of developing HTN compared to the first tertile (adjusted hazard ratio (HR), 1.21; 95% confidence interval (CI), 1.06, 1.37); by using Poisson regression models, Rezende-Alves et al. found that participants in the highest quintile of UPF energetic intake had the relative risk of 1.35 (95% CI, 1.01, 1.81) [4, 5]. Due to the high prevalence of HTN and different methods of processing food in different countries, it is crucial to assess the association of UPF/HTN in various populations, especially in the Middle East because of the limited available information regarding the consumption of UPF in the Middle Eastern people and how it could affect blood pressure in these populations. The Ravansar Non-Communicable Disease (RaNCD) cohort is the first cohort on the Iranian/Kurdish population in the western part of Iran; to obtain data regarding nutritional status, RaNCD uses a 113-item Food Frequency Questionnaire (FFQ); the main phase of the cohort was started in March 2015 with six completed follow-ups [6]. This study was conducted to fill the gap in our knowledge regarding UPF/HTN relations in the Middle East region.

## 2. Methods

### 2.1. Study Population

**Figure 1:**
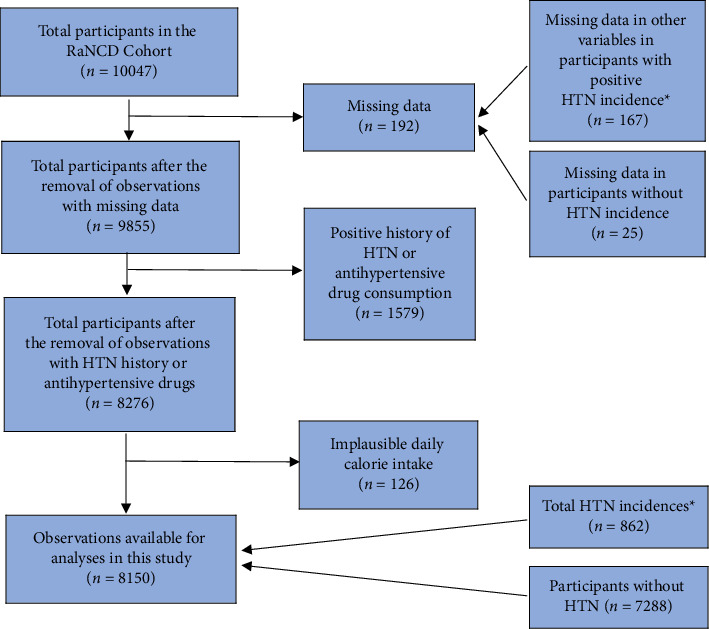
Flowchart of participants. Abbreviation: HTN, hypertension. ^∗^HTN was defined by systolic blood pressure (SBP) ≥ 140 mmHg and/or diastolic blood pressure (DBP) ≥ 90 mmHg in two or more readings by the cohort crew or other physicians; these instances were considered new incidences of HTN.

Utilizing data from the RaNCD cohort, we conducted a prospective analysis involving 10,047 participants aged 35–65 years who are permanently residing and have minimum residence of 1 year in urban or rural areas of Ravansar County, located in Kermanshah province; moreover, in total, 15,000 individuals aged 35–65 years were residing in both urban and rural areas of the Ravansar district; the researchers chose to include 10,000 of these individuals in the study who were willing to participate [6]. Firstly, we overviewed the overall data; we excluded 192 participants due to missing data; furthermore, 1579 participants had already been diagnosed with HTN or were using HTN drugs. Participants with improbable energy intake > 5500 kcal (*n* = 102) and < 800 kcal (*n* = 24) were also excluded. Finally, 8150 participants were incorporated in the final analysis (Figure 1).

### 2.2. Nutritional Status

Using the Iranian FFQ, 113 food groups and an additional four local foods were handed over to us for the investigation. We used the NOVA food classification system and designated each food group into one of the four NOVA groups (unprocessed, processed culinary ingredients, processed, and UPFs). The results were as follows: 66 food groups were classified as unprocessed, 12 as processed culinary ingredients, 24 as processed, and 19 as UPFs. We found 19 UPFs as follows: (1) baguette bread, (2) sausage/salami, (3) hamburger, (4) pizza, (5) flavored milk, (6) margarine, (7) hydrogenated oil, (8) mayonnaise, (9) rock candy/other sweets, (10) soda drinks, (11) nonalcoholic malt beverages, (12) ice cream, (13) dried cookies, (14) creamed cookies, (15) chocolate, (16) chips, (17) cheese puffs, (18) concentrated juice, and (19) crackers/biscuits (Table 1). All measurements were documented as gram/day (g/d).

### 2.3. HTN Incidence

HTN was defined as having a systolic blood pressure (SBP) ≥ 140 mmHg and/or a diastolic blood pressure (DBP) ≥ 90 mmHg based on two or more readings taken by the cohort crew or other physicians. As previously mentioned, in the initial phase of the cohort, 1579 participants were diagnosed with HTN or were using HTN-related drugs. New cases of HTN do not include those participants from the initial phase who were treated with antihypertensive medications or were previously diagnosed with HTN; consequently, all of these participants were excluded from the study. Participants were considered to have a new incidence of HTN after having an SBP ≥ 140 mmHg and/or a DBP ≥ 90 mmHg in two or more readings. In the follow-up phases, 1029 participants were identified as new incidence of HTN. After cleaning the data, we were left with 862 new incidences of HTN. The time at risk for the included participants was 22,774,803 days.

### 2.4. Statistical Analysis

We checked for several covariates including age, sex, residence types, marital status, socioeconomic status quartiles (SESq), history of fasting blood sugar (FBS), familial history (FH) of different diseases, metabolic equivalent (MET) quartiles, UPF tertiles, past medical history of different diseases, alcohol use, smoking status, total energy intake (Kcal/day), total carbohydrate intake (g/d), total lipid intake (g/d), and total protein intake (g/d). By adding up values of each UPF item (g/d), we estimated the total grams of UPF consumed in a day; then, we created tertiles of UPFs (tertile one had the least consumption, and tertile three had the highest amount). Cox proportional models were used to analyze data and estimate HRs and 95% CIs in the primary model and sensitivity analysis; *p* values below 0.05 (*p* < 0.05) are considered significant. Potential confounders for the main model were chosen based on the existing literature that commonly reported sex, age, residence type, marital status, SESq, FBS, first degree FH of HTN, and MET quartiles as confounders [4, 5].

To check whether the proportional hazards assumption is met in the Cox model, we used the proportional hazards test. After running the test, three variables, including age, residence, and the third tertile of UPF, violated the assumption. To address this problem, we introduced time-varying covariates into the main model.

To assess the robustness of our model, we designed several scenarios in the context of sensitivity analysis. First, we introduced alcohol consumption and smoking status to our model as further adjustments; in the next model, we further adjusted the model for total energy, carbohydrate, lipid, and protein intake in a day; in the following scenario, we brought in FHs to the model, namely, FHs of diabetes, cardiac diseases, myocardial infarction (MI), and stroke. In the next scenario, we excluded early cases (< 3 years) of HTN and executed the main model; next, we adjusted the main model for medical conditions, including rheumatoid arthritis (RA), osteoporosis, muscle weakness, and weight loss. Subgroup analyses were also performed; the purpose of the subgroup analyses in our study was to explore potential differences in the effects of UPF consumption across various demographics, namely, age (< 50 and > 50), sex (male and female), residence type (urban and rural), and clinical characteristics such as FH of HTN (negative and positive). Moreover, subgroup analysis is particularly important, as certain groups may be more susceptible to factors influencing HTN.

## 3. Results

### 3.1. Participant Characteristics

We have included 8150 participants in our study (47.58% male, 52.42% female), the mean age ± SD was 46.25 y ± 7.94, the mean follow-up time for HTN was 7.65 y ± 1.62, and the mean daily UPF intake in g/d among participants was 88.07 ± 84.96; approximately, half of participants had a positive FH of HTN (47.82%), and the mean BMI was 27.32 ± 4.61; Table 2 shows characteristics of participants in each UPF tertile. RaNCD participants in the third tertile of UPF consumption are more likely to be men, younger, live in the city, be smokers, have better SES, consume more energy (fourth quantile of energy intake), and have almost the same physical activity and BMI compared to the first tertile.

### 3.2. Main Model

We used the Cox proportional hazard model (Table 3) as our main model, and to assess how sensitive our primary model is. Covariates included in the main model were as follows: age, sex, BMI, residence type, marital status, socioeconomic status, physical activity, FH of HTN, and FBS. We followed a dual approach regarding BMI. Table 3A shows the results of the main model without including BMI as a confounding factor; moreover, Table 3B shows the results when we included BMI in the main model. A 1-unit increase in BMI corresponds to a 6% increase in the chance of developing HTN, with an HR of 1.06 (95% CI, 1.04–1.07; *p* value < 0.001). The HR and 95% CI of the second and third tertile of UPFs were 1.13 (95% CI, 0.96–1.32) and 0.65 (95% CI, 0.46–0.91; *p* value = 0.01), respectively, compared to the first tertile.

### 3.3. Sensitivity Analysis

**Figure 2:**
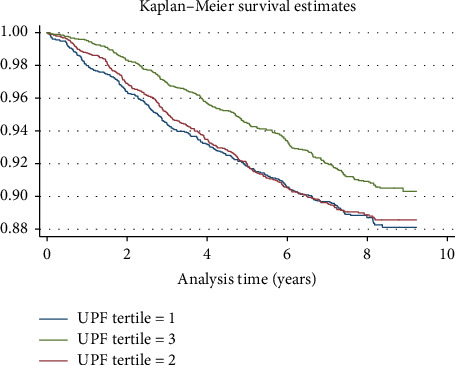
The Kaplan–Meier curve stratified by UPF tertiles; the *x*-axis is measured in years.

Several scenarios as sensitivity analyses were designed to test our main model's robustness. Initially, we further adjusted the main model; the adjustments made included alcohol use, smoking status, total lipid intake, total carbohydrate intake, total protein intake, total energy consumption, family histories of noncommunicable diseases (NCDs) such as type 2 diabetes, cardiac diseases, MI, and stroke, as well as PMHs including RA, osteoporosis, and gastroesophageal reflux disease (GERD). None of these factors showed significant results, except for a familial history of stroke, and the HR was 1.28 (95% CI, 1.07–1.54, *p* value = 0.007). Consequently, we excluded early cases of HTN incidence (< 3 y), and the results did not change compared to the main model. In the following scenario, we checked for interactions; the interaction between sex and BMI showed HR of 0.94 (0.91–0.97, 0.000), meaning that the impact of BMI on HTN incidence in females is approximately 6% less than males. The results of the subgroup analysis can be found in Table 4. Furthermore, the probability of survival during the analysis period for each UPF tertile, as estimated by the Kaplan–Meier curve, can be found in Figure 2.

Log-rank tests were also performed and they showed significant differences in HTN incidence based on sex (chi2 = 41.98, *p* < 0.001), SESq (chi2 = 18.86, *p* < 0.001), MET quantiles (chi2 = 21.12, *p* < 0.001), and UPF tertiles (chi2 = 9.53, *p* < 0.001) (Table 5). No significant differences were observed for residence type, marital status, or FH of HTN.

## 4. Discussion

In this study, we aimed to answer the question of whether there is a relationship between UPF consumption and HTN incidence in Middle Eastern populations or there is not; moreover, 8150 participants were included, and they were followed for an average of 7.65 years. Our participants were mostly female (52.42%), and the mean age was 46.25 years. Our findings showed that increased UPF consumption significantly reduced the HR for HTN incidence in the third UPF tertile (0.65; 95% CI: 0.46–0.91, *p* value = 0.01). The third tertile interacted with time in our model; therefore, the result should be interpreted cautiously. The HR for the second tertile of UPF consumption was not significant (1.13; 95% CI: 0.96–1.32, *p* value = 0.13) compared to the first tertile.

To the best of our knowledge, our study is the first to assess the HTN/UPF associations in the Middle East and North Africa region with participants that had diverse backgrounds in different aspects. We previously converted the Persian FFQ used in the RaNCD cohort into NOVA groups; the results were as follows: unprocessed (59.5%), processed culinary (10.2%), processed (27.1%), and UPFs (3.0%). Several hypotheses can be made to justify our findings; firstly, the consumption of UPFs in our participants was extremely low (3.0%) compared to other studies; Martinez-Perez et al. reported that the mean UPF consumption in grams per day was 7.9% [7]; Monge et al. and da Silva Scaranni et al. measured UPF consumption percentages as total energy consumed and found that UPF contributions were 29.8% and 25.2%, respectively [8, 9]. Our results suggest that consuming UPFs in small quantities may not be associated with HTN incidence and even it might have an opposite effect. Secondly, we strictly adjusted covariates and tried to be cautious about categorizing controversial food items as UPFs. Previous studies had reported mixed results regarding UPFs/HTN association; some found a positive association; for example, Mendonça et al. used the Cox proportional regression model as their primary model and found participants in the last tertile of UPF consumption had adjusted HR of 1.21 (95% CI, 1.06 - 1.37, p-for-trend = 0.004); others used a wide range of statistical models including Poisson regression, linear regression, and *t*-test and found no significant associations [7, 8, 10]. After accounting for temporal aspects of HTN incidence (HRs), we found that females have 47% more chance to develop HTN compared to males; we also found that 1 year increase in age corresponds to 9% higher chance of HTN incidence. Looking at familial aspects of HTN incidence, we found that a positive HTN history in first-degree relatives is associated with a 28% higher chance of HTN incidence. Participants in the highest MET quantile had lower chance of developing HTN by 24%.

Our study, while comprehensive, did face some limitations. The Persian FFQ, while not specifically designed for determining NOVA food groups, was manually determined by our team. We also encountered instances of missing data, and we chose to analyze the records with complete data. Despite our best efforts to adjust the model for all relevant confounders, there may be some unidentified ones. Lastly, longer follow-up periods could potentially reveal different results. These limitations, while present, do not diminish the robustness of our research.

Our study has some substantial strengths as well. We designed several sensitivity analysis scenarios to assess the integrity of the main model; furthermore, our cohort participants had a diverse background, and they did not belong to a specific group; diverse backgrounds of participants add to the generalization of our results.

In conclusion, our study, conducted using Cox proportional hazard models, did find a significant association between HTN Incidence and the third tertile UPF consumption. These findings, while not definitive, are significant and call for further studies with longer time frames and more participants to verify and expand upon our results.

## Figures and Tables

**Table 1:** NOVA classification of Persian cohort FFQ items.

*Group 1—unprocessed or minimally processed foods*
Milk, tea, egg (boiled), mushroom, sweet cherry, banana, fresh figs, apricot, sour cherry, cantaloupe melon, peach, plums, persimmon, grapes, dates, olives, apple, kiwi, watermelon, nectarine, honeydew, fresh berries, strawberries, pomegranate, pears, citrus fruits, dried fruits, raisin, fresh leafy greens, cabbage, tomato, zucchini, cucumber, potato, carrot, garlic, onion, beets, lettuce, green beans, green peas, green peppers, bell peppers, celery, eggplant, soybean, peanut, walnut, seeds, other nuts, boiled or grilled red meat, a variety of meats and their products, boiled chicken, boiled sheep brain, boiled sheep tongue, chicken giblet, boiled or grilled fish, corn, wheat, wheat oats barley, boiled chickpeas, boiled split peas, boiled lentils, boiled fava beans.

*Group 2—processed culinary ingredients*
Olive oils, other plant oils, butter, honey, salt, cooked rice, cooked leafy greens, cooked traditional greens, white sugar, sugar cube, fruit juice.

*Group 3—processed foods*
Cheese, yoghurt, dough, kashk, clotted cream, Kermanshahi ghee, pasta noodles, lavash bread, barbary bread, sangak bread, barely bread, traditional bread, pickles, torshi, pickled vegetables, jam, tomato pastes, pomegranate paste, tuna canned, compotes, tahini halva, NESCAFE coffee.

*Group 4—ultra-processed foods (% * ^*∗*^ * )*
Margarine (0.95%), hydrogenated oils (26.7%), baguette bread (0.79%), hamburger (0.76%), pizza (1.06%), sausages or bologna (0.35), soda drinks (16.11%), nonalcoholic beverages malt (10.84%), flavored milk (4.56%), ice cream (5.12%), mayonnaise (1.44%), concentrate juice (8.81%), candies or sweets (1.79%), chips (1.26%), cheese puffs (1.68%), crackers or biscuits (5.47%), dried cookies (7.23%), creamed cookies (2.38%), chocolate (2.6%).

^∗^The mean grams consumed of each ultra-processed food divided by the mean total grams of ultra-processed foods, based on percentage.

**Table 2:** Characteristics of participants in each UPF tertile.

Characteristics	Tertile 1 (*N* = 2735)	Tertile (*N* = 2746)	Tertile 3 (*N* = 2669)	*p* values
	Mean ± SD or N (percentage)			
UPF intake (g/d)	24.53 ± 11.58	63.83 ± 13.80	178.13 ± 94.11	< 0.001
Age (years)	47.87 ± 8.08	46.27 ± 7.93	44.58 ± 7.46	< 0.001
Sex				< 0.001
Male	969 (35.42)	1274 (46.39)	1635 (61.25)	
Marital status				< 0.001
Single	143 (5.22)	141 (5.13)	100 (3.74)	
Residence type				< 0.001
Urban	1425 (52.10)	1536 (55.93)	1891 (70.85)	
Alcohol use				< 0.001
No	2689 (98.31)	2627 (95.66)	2438 (91.34)	
BMI	27.49 ± 4.61	27.11 ± 4.56	27.36 ± 4.65	0.0055
Smoking				< 0.001
No	1253 (45.81)	1177 (42.86)	936 (35.06)	
Current	222 (8.11)	312 (11.36)	442 (16.56)	
Former	202 (7.38)	222 (8.08)	206 (7.71)	
Passive	1058 (38.68)	1035 (37.69)	1085 (40.65)	
SES quantiles				< 0.001
1	682 (24.94)	572 (20.83)	315 (11.80)	
2	550 (20.11)	566 (20.61)	482 (18.06)	
3	532 (19.45)	518 (18.86)	602 (22.56)	
4	479 (17.51)	542 (19.74)	621 (23.27)	
5	492 (17.99)	548 (19.96)	649 (24.32)	
T2DM				< 0.001
Yes	209 (48.37)	125 (28.93)	98 (22.68)	
CVDs				< 0.001
Yes	232 (8.48)	170 (6.19)	124 (4.65)	
MET				< 0.001
1	650 (23.77)	703 (25.60)	700 (26.23)	
2	770 (28.15)	657 (23.93)	610 (22.86)	
3	760 (27.79)	689 (25.09)	613 (22.97)	
4	555 (20.29)	697 (25.38)	746 (27.95)	
HTN (FH)				0.318
Yes	1280 (46.80)	1313 (47.82)	1304 (48.86)	
Energy (kcal)	2224.68 ± 723.68	2620.77 ± 760.52	3248.80 ± 867.31	< 0.001

*Note:p* values for categorical variables were calculated using the chi-squared test, and for continuous variables, the Kruskal–Wallis test was employed.

**Table 3:** The summary of the main model.

**A**	**HR.**	**p** **value**	**(95% conf interval)**	

Sex (1 = male, 2 = female)	1	—	—	—
2 (female)	1.47	< 0.001	1.25	1.74
Age	1.09	< 0.001	1.08	1.11
Residence (city = 1 rural = 2)	1	—	—	—
2 (rural)	1.17	0.27	0.87	1.57
Marital status (1 = single 2 = Married)	1	—	—	—
2 (married)	1.24	0.06	0.98	1.57
5 quantiles of SES	1	—	—	—
2	1.21	0.07	0.98	1.48
3	1.10	0.38	0.88	1.37
4	1.04	0.73	0.82	1.31
5	1.01	0.90	0.78	1.32
FBS	1.003	0.001	1.001	1.005
FH_HTN No	1	—	—	—
Yes	1.28	< 0.001	1.11	1.46
4 quantiles of MET	1	—	—	—
2	0.89	0.26	0.74	1.08
3	1.04	0.61	0.87	1.26
4	0.76	0.01	0.61	0.94
3 quantiles of UPFs	1	—	—	—
2	1.13	0.13	0.96	1.32
3	0.65	0.01	0.46	0.91
TVC^∗^ (time-varying covariates)				
Age	0.99	< 0.001	0.99996	0.99998
Residence (rural)	0.99	0.043	0.99961	0.99999
Third tertile of UPF	1.00	0.001	1.00012	1.00051

**B**	**HR.**	**p** **value**	**(95% conf interval)**	

Sex (1 = male, 2 = female)	1	—	—	—
2 (female)	1.24	0.012	1.04	1.47
Age	1.10	< 0.001	1.08	1.12
Residence (city = 1 rural = 2)	1	—	—	—
2 (rural)	1.24	0.13	0.93	1.67
Marital status (1 = single 2 = Married)	1	—	—	—
2 (married)	1.17	0.18	0.92	1.48
5 quantiles of SES	1	—	—	—
2	1.16	0.14	0.94	1.43
3	1.05	0.64	0.84	1.31
4	0.98	0.89	0.77	1.24
5	0.97	0.84	0.74	1.26
FBS	1.002	0.007	1.000	1.004
FH_HTN No	1	—	—	—
Yes	1.24	0.002	1.08	1.42
4 quantiles of MET	1	—	—	—
2	0.92	0.40	0.76	1.11
3	1.09	0.33	0.90	1.31
4	0.81	0.06	0.66	1.01
3 quantiles of UPFs	1	—	—	—
2	1.14	0.09	0.97	1.34
3	0.64	0.01	0.46	0.90
BMI	1.06	< 0.001	1.04	1.07
TVC^∗^ (time-varying covariates)				
Age	0.99	< 0.001	0.99996	0.99998
Residence (rural)	0.99	0.038	0.99960	0.99998
Third tertile of UPF	1.00	0.001	1.00012	1.00050

*Note:* A without considering BMI as a confounder, B including BMI.

^∗^Variables in TVC equation interacted with time.

**Table 4:** HRs and *p* values of age, sex (females compared to males), residence (rural compared to urban), and each UPF tertile in the subgroup analysis.

Subgroups	UPF tertiles						Sex		Age		Residence	
	Tertile 1		Tertile 2		Tertile 3							
	HR	*p* value	HR (95% CI)	*p* value	HR (95% CI)	*p* value	HR (95% CI)	*p* value	HR (95% CI)	*p* value	HR (95% CI)	*p* value
Age > 50	1.00	—	1.08 (0.86, 1.36)	0.474	0.95 (0.72, 1.24)	0.719	1.35 (1.05, 1.73)	0.016	1.01 (0.98, 1.04)	0.280	0.94 (0.73, 1.19)	0.619
Age < 50	1.00	—	1.13 (0.90, 1.43)	0.278	1.10 (0.86, 1.41)	0.415	1.59 (1.27, 2.01)	< 0.001	1.10 (1.08, 1.13)	< 0.001	0.93 (0.75, 1.17)	0.573
Males	1.00	—	1.08 (0.82, 1.43)	0.545	0.88 (0.66, 1.16)	0.380	—	—	1.06 (1.05, 1.08)	< 0.001	0.93 (0.72, 1.20)	0.618
Females	1.00	—	1.13 (0.93, 1.38)	0.205	1.17 (0.93, 1.46)	0.161	—	—	1.04 (1.03, 1.05)	< 0.001	0.94 (0.76, 1.15)	0.556
Urban	1.00	—	1.03 (0.84, 1.28)	0.718	0.95 (0.77, 1.18)	0.687	1.44 (1.17, 1.78)	0.001	1.05 (1.04, 1.06)	< 0.001	—	—
Rural	1.00	—	1.26 (0.98, 1.62)	0.062	1.17 (0.86, 1.61)	0.301	1.59 (1.21, 2.08)	0.001	1.05 (1.03, 1.06)	< 0.001	—	—
FH HTN												
Yes	1.00	—	1.34 (1.07, 1.68)	0.010	1.12 (0.88, 1.44)	0.338	1.45 (1.15, 1.85)	0.002	1.04 (1.03, 1.06)	< 0.001	1.04 (0.83, 1.31)	0.680
No	1.00	—	0.94 (0.75, 1.19)	0.659	0.95 (0.74, 1.22)	0.697	1.51 (1.19, 1.90)	0.001	1.05 (1.04, 1.07)	< 0.001	0.82 (0.66, 1.03)	0.096

**Table 5:** Log-rank test results.

Log-rank test results	chi2	*p* value
Sex	41.98	< 0.001
Residence	0.57	0.47
Marital status	0.01	0.90
SES quantiles	18.86	< 0.001
FH_HTN	3.66	0.055
MET quantiles	21.12	< 0.001
UPF tertile	9.53	< 0.001

## Data Availability

The datasets generated and/or analyzed during the current study are not publicly available but are available from the corresponding author on reasonable request.

## References

[1] Monteiro C. A., Cannon G., Levy R. B. (2019). Ultra-processed Foods: What They Are and How to Identify Them. *Public Health Nutrition*.

[2] Baker P., Machado P., Santos T. (2020). Ultra‐processed Foods and the Nutrition Transition: Global, Regional and National Trends, Food Systems Transformations and Political Economy Drivers. *Obesity Reviews: An Official Journal of the International Association for the Study of Obesity*.

[3] World Health Organization (2024). Hypertension. https://www.who.int/news-room/fact-sheets/detail/hypertension.

[4] Mendonça R. d D., Lopes A. C., Pimenta A. M., Gea A., Martinez-Gonzalez M. A., Bes-Rastrollo M. (2017). Ultra-processed food consumption and the incidence of hypertension in a Mediterranean cohort: the Seguimiento Universidad de Navarra Project. *American Journal of Hypertension*.

[5] Rezende-Alves K., Hermsdorff H. H., Miranda A. E. d S., Lopes A. C., Bressan J., Pimenta A. M. (2021). Food Processing and Risk of Hypertension: Cohort of Universities of Minas Gerais, Brazil (CUME Project). *Public Health Nutrition*.

[6] Pasdar Y., Najafi F., Moradinazar M. (2019). Cohort Profile: Ravansar Non-communicable Disease Cohort Study: the First Cohort Study in a Kurdish Population. *International Journal of Epidemiology*.

[7] Martinez-Perez C., San-Cristobal R., Guallar-Castillon P. (2021). Use of Different Food Classification Systems to Assess the Association between Ultra-processed Food Consumption and Cardiometabolic Health in an Elderly Population with Metabolic Syndrome (PREDIMED-Plus Cohort). *Nutrients*.

[8] Monge A., Silva Canella D., López-Olmedo N., Lajous M., Cortés-Valencia A., Stern D. (2021). Ultraprocessed Beverages and Processed Meats Increase the Incidence of Hypertension in Mexican Women. *British Journal of Nutrition*.

[9] Scaranni P. d O. d S., Cardoso L. d O., Chor D. (2021). Ultra-processed Foods, Changes in Blood Pressure and Incidence of Hypertension: the Brazilian Longitudinal Study of Adult Health (ELSA-Brasil). *Public Health Nutrition*.

[10] da Conceiįão A. R., de Almeida Fonseca P. C., de Castro Morais D., de Souza E. C. (2019). Association of the Degree of Food Processing With the Consumption of Nutrients and Blood Pressure. *O Mundo da Saúde*.

[11] Amirian P., Zarpoosh M., Pasdar Y. (2024). Ultra-processed Foods and Hypertension Incidence in RaNCD Cohort Project. https://www.medrxiv.org/content/10.1101/2024.06.13.24308899v1.full.

